# Challenges in overcoming advanced-stage or relapsed refractory extranodal NK/T-cell lymphoma: meta-analysis of individual patient data

**DOI:** 10.3389/fonc.2024.1362367

**Published:** 2024-07-31

**Authors:** Tong Yoon Kim, Tae Jung Kim, Eun Ji Han, Gi June Min, Youngwoo Jeon, Seok-Goo Cho

**Affiliations:** ^1^ Department of Hematology, Yeouido St. Mary’s Hospital, College of Medicine, The Catholic University of Korea, Seoul, Republic of Korea; ^2^ Lymphoma and Cell Therapy Research Center, Yeouido St. Mary’s Hospital, College of Medicine, The Catholic University of Korea, Seoul, Republic of Korea; ^3^ Department of Hospital Pathology, Yeouido St. Mary’s Hospital, College of Medicine, The Catholic University of Korea, Seoul, Republic of Korea; ^4^ Division of Nuclear Medicine, Department of Radiology, Yeouido St. Mary’s Hospital, College of Medicine, The Catholic University of Korea, Seoul, Republic of Korea; ^5^ Department of Hematology, Seoul St. Mary’s Hospital, College of Medicine, The Catholic University of Korea, Seoul, Republic of Korea

**Keywords:** extranodal NK/T-cell lymphoma, advanced, relapsed/refractory, estimate individual patient data, meta-analysis, Epstein–Barr virus

## Abstract

**Introduction:**

Extranodal NK/T-cell lymphoma (ENKTCL), a non-Hodgkin lymphoma, is known for its destructive local impact on nasal structures and systemic induction of inflammatory cytokines. Concurrent treatment with radiation and nonanthracycline- based chemotherapy has improved survival rates in patients with localized disease stages. However, survival outcomes vary significantly in advanced-stage and relapsed or refractory (R/R) cases.

**Methods:**

Therefore, we conducted a meta-analysis using random effects models to assess prognostic factors in advanced or R/R ENKTCL, employing a digital extractor on Kaplan–Meier graphs owing to the scarcity of published prospective trials for these patients.

**Results:**

We observed that patients with advanced ENKTCL treated with Lasparaginase had a median progression-free survival (PFS) of 14.3 months and an overall survival (OS) of 19 months. In R/R ENKTCL, PFS and OS were 11.7 and 15.6 months, respectively. Additionally, OS outcomes in advanced-stage ENKTCL were better in the asparaginase group than that in the non-asparaginase group, with PEG-asparaginase showing superior results compared with that using Lasparaginase. Epstein–Barr Virus (EBV)-DNA positivity in the bloodstream prior to treatment was associated with poor outcomes in advanced-stage ENKTCL, and similar trends were observed in patients with R/R ENKTCL and post-treatment EBV viremia.

**Discussion:**

Collectively, these findings suggest that chemotherapy with Lasparaginase or PEG-asparaginase can enhance survival in advanced or R/R ENKTCL. However, future strategies must be developed to effectively suppress EBV viremia and achieve a deep response toward tumor eradication.

## 1 Introduction

Patients with extranodal NK/T-cell lymphoma (ENKTCL) display distinctive characteristics compared with those with other non-Hodgkin cell lymphomas. ENKTCL primarily affects the nasal mucosa, leading to destruction of adjacent structures, including the nasopharynx, oropharynx, oral cavity, and hypopharynx. Notably, 72.4–75% of cases are diagnosed at stages I/II, with systemic spread being uncommon (1, 2).

Survival outcomes for patients with Ann Arbor stages I/II ENKTCL have improved substantially. Notably, the 5-year overall survival (OS) rates increased from 38% to 42% (3, 4) in the early 2000s, whereas 3-year OS rates have escalated to 85% in the last two decades (5, 6). These improvements are attributed to the administration of radiation therapy exceeding 50 Gy, complemented by non-anthracycline-based chemotherapy regimens. These regimens typically include agents such as etoposide, gemcitabine, ifosfamide, methotrexate, and platinum, employed as first-line therapies. The 5-year OS rates for patients with advanced-stage ENKTCL display significant variability, ranging from 30–74.3% (3, 7, 8). This variation is attributed to the use of L-asparaginase (L-Asp) or PEG-asparaginase (PEG-Asp), which typically result in improved outcomes. However, some studies report lower survival rates, specifically between 33.2–45.7%, when incorporating L-Asp (9, 10). Similarly, survival in relapsed or refractory (R/R) ENKTCL is inconsistent, with 5-year OS rates ranging from 24.8–55% (11, 12).

In this study, we integrated studies on advanced and R/R ENKTCL to estimate OS and progression-free survival (PFS) using random effects models for survival curve synthesis. Our meta-analysis, incorporating hazard ratios for OS, was conducted to discern the impact of various factors on survival outcomes, particularly comparing L-Asp and PEG-Asp treatments and the presence or absence of Epstein–Barr Virus (EBV) DNAemia.

Differences in results stem from heterogeneity in cohort subgroup analyses and the inclusion of relatively small patient groups; to address this, we utilized individual patient data (IPD) from published graphs and conducted an IPD meta-analysis to identify potential targets for future treatments of advanced or R/R ENKTCL.

## 2 Methods

### 2.1 Data retrieval and search strategy

Web-based retrieval was conducted manually through PubMed (January 2000 to November 2023) and Embase (January 2000 to November 2023), using the search term ENKTCL. Adult patients with newly diagnosed advanced-stage R/R ENKTCL were enrolled in phase I, II, and III clinical trials as well as retrospective studies. Two researchers (T.K. and G.M.) independently screened articles that met the eligibility criteria and extracted data from the literature.

### 2.2 Study selection criteria and quality evaluation

Studies were analyzed using the following inclusion criteria: patients treated with chemotherapy regimens; reported survival data or curves; and published in English. The search included the keywords “extranodal natural killer T-cell lymphoma and chemotherapy.” The quality evaluation of the analyzed studies complied with the Cochrane Handbook for Systematic Reviews (Version 5.1.0) (13).

We selected studies that included data on OS and PFS for stages III and IV, or R/R ENKTCL. To analyze the factors that impact OS, we analyzed studies that presented data categorized by treatment with PEG-Asp and Asp. To evaluate the EBV status in the advanced-stage ENTKCL, we specifically focused on studies discussing EBV DNA before treatment and in cases of relapsed ENKTCL. Additionally, studies indicating post-treatment EBV positivity were included.

### 2.3 Data extraction and analysis

For the estimation of PFS and OS, we utilized IPD from the Kaplan–Meier (KM) package were used in patients with advanced-stage or R/R ENKTCL (14).. Unavailable or missing data were imputed using the graph and were reconstructed using ScanIt software (https://www.amsterchem.com/scanit.html). To ascertain factors affecting OS, we extracted results that included hazard ratios (HRs) and 95% confidence intervals (CIs). HRs were calculated to interpret prognostic factors, and a meta-analysis was performed using the meta and metafor packages (15, 16). A *p*-value < 0.05 indicated statistical significance. The heterogeneity test in these studies was considered statistically significant with a p-value <0.10. I^2^ was employed for quantitative analysis of heterogeneity: I^2^ < 25% indicated low heterogeneity, 25% ≤ I^2^ ≤ 50% suggested moderate heterogeneity, and I^2^ > 50% indicated high heterogeneity. The random effects model was employed to compare the HR and 95% CIs. Statistical analysis was performed using R software for statistical computing (R Foundation for Statistical Computing, Vienna, Austria, version 4.0.2).

## 3 Results

### 3.1 Retrieved results and characteristics of included studies

**Figure 1:**
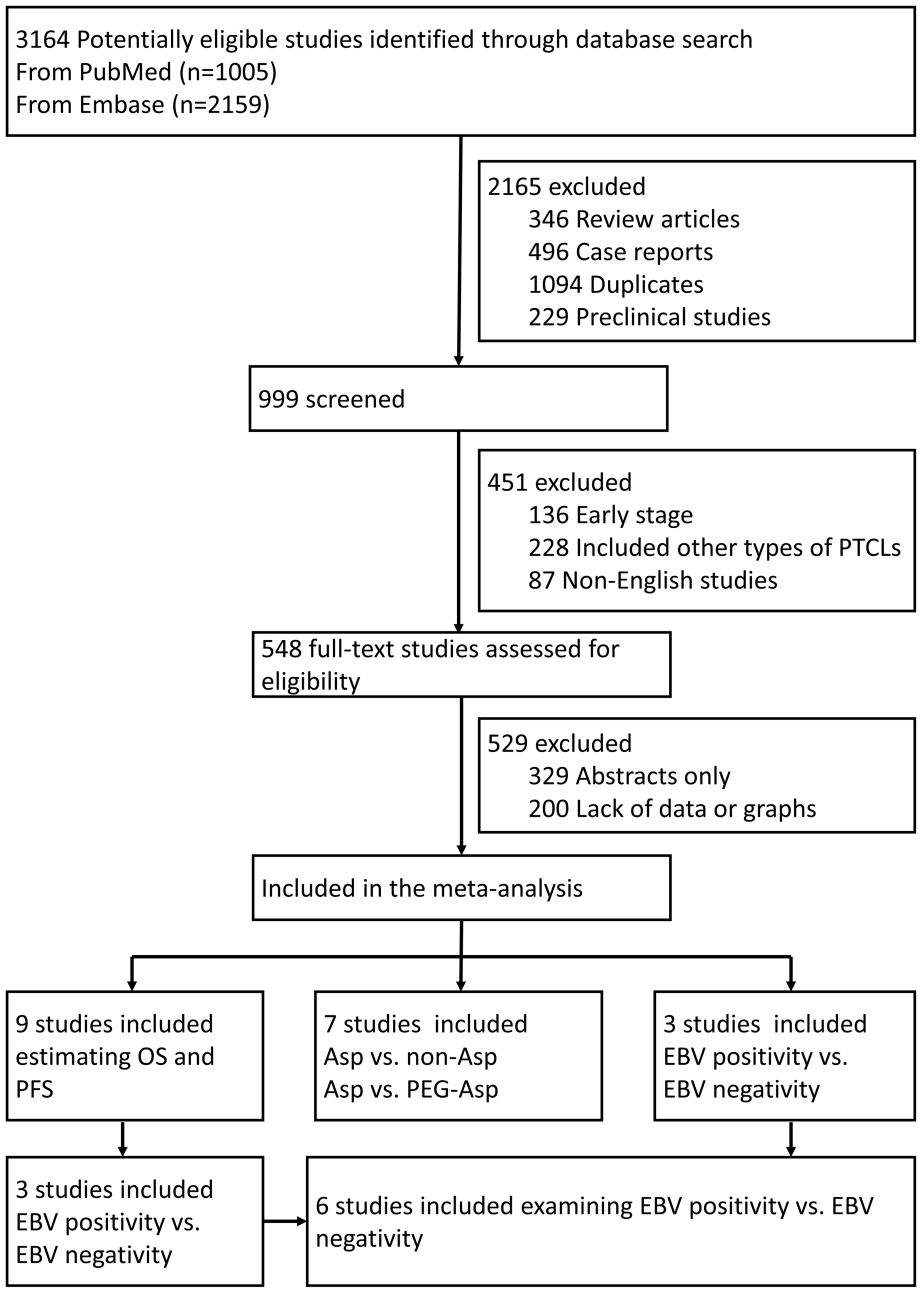
Flowchart of the trial selection process. PTCLs, Peripheral T-Cell Lymphoma; OS, overall survival; PFS, progression-free survival; Asp, L-asparaginases; EBV, Epstein–Barr Virus.

According to the retrieval strategy, 3164 related references were checked. Among these, 548 were preliminarily screened after reading the title and abstract, excluding repetitive nonclinical studies and literature unrelated to treatments. Eventually, 19 studies were selected after reading the full articles (**Figure 1**).

### 3.2 Quality evaluation of included studies

All included studies provided comprehensive details on patient conditions and complete result data but lacked detailed descriptions of randomization, blinding methods, follow-up losses, and allocation concealment. Consequently, the overall evaluation rate was relatively low. No significant publication bias was observed in the comparison of L-Asp versus non-Asp-based chemotherapy and PEG-Asp versus Asp-based chemotherapy in patients with advanced-stage ENKTCL. The same applies to elevated versus normal EBV DNA in pretreatment blood in stages I–IV ENKTCL patients (**Supplementary Figures S1A–C**). However, the comparison between elevated (over 500 copies) and normal EBV DNA in end-of-treatment blood in patients with R/R ENKTCL deviated from the expected plot. This deviation is likely attributed to the limited number of studies and their heterogeneity (**Supplementary Figure S1D**).

### 3.3 Estimating PFS and OS in patients with advanced-stage or R/R ENKTCL

**Figure 2:**
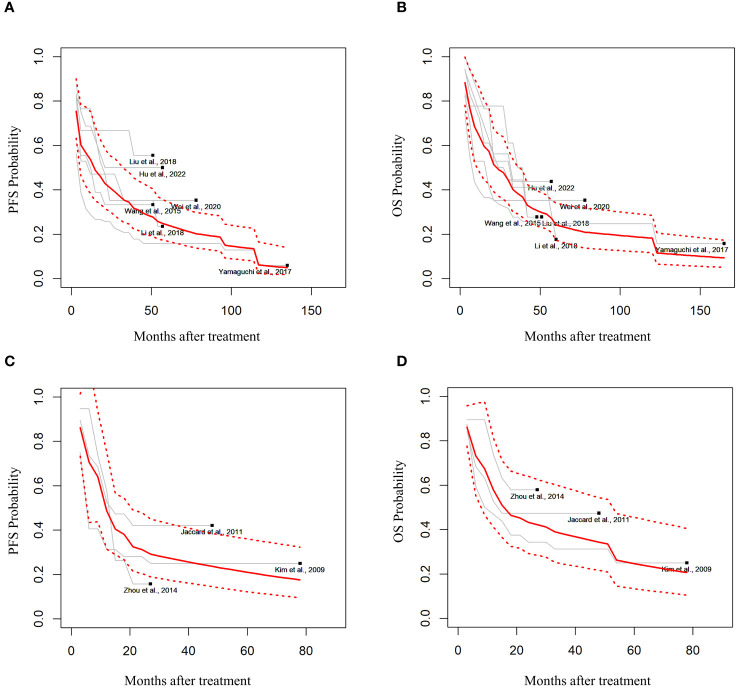
Estimated survival outcomes in advanced-stage and relapsed/refractory (R/R) ENKTCL. **(A)** Progression-free survival (PFS) and **(B)** overall survival (OS) in patients with stage III or IV ENKTCL. **(C)** PFS and **(D)** OS in patients with R/R ENKTCL.

Among the 19 studies, six (9, 10, 17–20) extracted the IPD using graphs (**Table 1**). In the random effects model, 188 patients with advanced-stage III–IV ENKTCL treated with L- or PEG-Asp-based therapy showed an estimated median PFS of 14.3 months (95% CI not applicable; in the fixed-effects model: 9 months, 95% CI, 5.26–6.07) and an OS of 19 months (95% CI, 11.26–27.36) (**Figures 2A, B**).

**Table 1:** Baseline characteristics of trials analyzing patients with advanced-stage or relapsed/refractory (R/R) ENKTCL.

Category	First author	Year	Phase	Treatment	No. of patients	Median age (range)	Male	Stage III/IV	EBV (+)	EBV (–)	Ref
Stage III or IV ENKTCL PFS and OS	Wang et al.	2015	Retro	LVDP	18	35 (16–63)	9	18	no data	no data	(17)
Stage III or IV ENKTCL PFS and OS	Yamaguchi et al.	2017	Retro	SMILE	358	58 (16–88)	240	101	no data	no data	(18)
Stage III or IV ENKTCL PFS, OS and EBV status	Li et al.	2018	retro	GELOXD or P-GEMOXD	184	43 (10–76)	131	17	38	30	(19)
Stage III or IV ENKTCL PFS and OS and EBV status	Liu et al.	2018	retro	DDGP	57	no data	36	18	33	24	(20)
Stage III or IV ENKTCL PFS and OS	Wei et al.	2020	II	GDPML	44	44 (24–68)	30	18	29	15	(9)
Stage III or IV ENKTCL PFS and OS	Hu et al.	2022	II	COEPL	80	41 (15–76)	57	16	21	22	(10)
R/R ENKTCL PFS and OS	Kim et al.	2009	retro	IMVP	32	45 (23–65)	18	17	no data	no data	(11)
R/R ENKTCL PFS, OS and EBV status	Jaccard et al.	2011	II	AspMetdex	19	60 (45–76)	15	7	7	5	(21)
R/R ENKTCL PFS and OS	Zhou et al.	2014	retro	DDGP	17	42 (13–65)	6	9	no data	no data	(22)
Asp vs. non-Asp	Kim et al.	2015	retro	IMEPL vs. non-Asp	70	48.5 (18–73)	48	70	no data	no data	(23)
Asp vs. non-Asp	Li et al.	2020	retro	Asp vs. CHOP	107	42 (10–76)	75	107	no data	no data	(24)
Asp vs. non-Asp	Liu et al.	2021	retro	Asp vs non-Asp	336	42 (6–84)	238	336	no data	no data	(25)
Asp vs. non-Asp	Wei et al.	2023	retro	Asp vs non-Asp	195	43 (6–84)	136	195	48	60	(26)
PEG-Asp vs. Asp	Li et al.	2016	I	DDGP vs. SMILE	42	42 (14–64)	26	42	no data	no data	(27)
PEG-Asp vs. Asp	Wei et al.	2020	III	SVILE vs. PGEMOX	103	46 (18–67)	68	34			(28)
PEG-Asp vs. Asp	Wang et al.	2022	II	DDGP vs. SMILE	80	42 (6–84)	51	80	39	51	(7)
Stage III or IV ENKTCL EBV status	Liang et al.	2017	retro	PEMD	32	48 (17–73)	25	32	13	9	(29)
R/R ENKTCL EBV status	Wang et al.	2021	retro	DDGP vs. SMILE	54	39 (15–65)	38	38	24	30	(30)
R/R ENKTCL EBV status	Huang et al.	2021	II	Daratumumab	32	56 (22–78)	23	no data	23	8	(31)

No, number; EBV, Epstein–Barr Virus; Ref, reference; OS, overall survival; PFS, progression-free survival; Retro, retrospective study; Asp, L-asparaginases; LVDP, L-asparaginase, etoposide, dexamethasone, and cisplatin; SMILE, dexamethasone, methotrexate, ifosfamide, L-asparaginase, and etoposide; GELOXD, gemcitabine, oxaliplatin, L- asparaginases, and dexamethasone; P-GEMOXD, gemcitabine, oxaliplatin, PEG-asparaginase, and dexamethasone; DDGP, dexamethasone, cisplatin, gemcitabine, and pegaspargase; GDPML, gemcitabine, cisplatin, dexamethasone, methotrexate, and pegaspargase; COEPL, pegaspargase, cyclophosphamide, vincristine, etoposide, and prednisone; IMVP, etoposide, ifosfamide, methotrexate, and prednisolone; AspaMetDex, L-asparaginase, methotrexate, and dexamethasone; IMEPL, Ifosfamide, methotrexate, etoposide, and prednisolone and L-asparaginase; PEMD, PEG-asparaginase, etoposide, methotrexate, and dexamethasone.

Three studies had estimated the survival outcomes in patients with R/R ENKTCL (11, 21, 22). Among 68 patients with R/R ENKTCL who were treated with L- or PEG-Asp-based treatment in random effects, the median PFS was estimated to be 11.7 months (95% CI, 4.76–16.43), and OS was 15.6 months (95% CI, 5.97–33.56) (**Figures 2C, D**).

### 3.4 Factors influencing OS in advanced-stage or R/R ENKTCL

Patients with stages III–IV or R/R ENKTCL were divided into Asp, non-Asp, EBV-negative, and EBV-positive groups. **Table 1** summarizes the characteristics of the included studies. The HR evaluation of the 13 studies focused on four comparisons: Asp group versus non-Asp group, Asp group versus PEG-Asp group, and EBV DNA-negative group versus EBV DNA-positive group.

**Figure 3:**
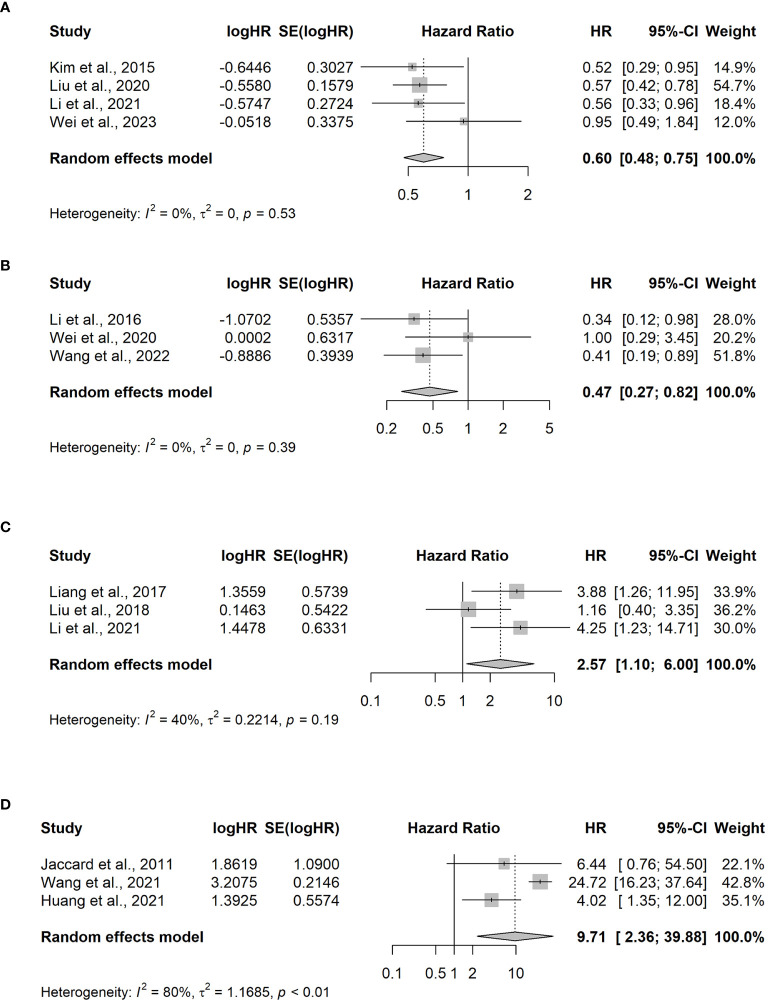
Meta-analysis of hazard ratios for factors impacting overall survival. **(A)** L-asparaginase (Asp) versus non-Asp-based chemotherapy; **(B)** PEG-asparaginase versus Asp-based chemotherapy in advanced-stage ENKTCL patients; **(C)** elevated versus normal EBV DNA levels in the blood pretreatment for III–V ENKTCL; **(D)** elevated versus normal EBV DNA levels in the blood at the end of treatment for relapsed/refractory ENKTCL. CI, confidence interval; HR, hazard ratio.

Among the 13 studies, four of them (23–26) compared the OS benefit between the Asp and non-Asp groups. A total of 481 patients, including 268 and 213 cases in the Asp and non-Asp group, respectively, were included in the analysis, and no statistical heterogeneity was observed (*p* = 0.529, I^2^ = 0%). Combined analysis results indicated a significant difference in HR between the groups [HR = 0.60, 95% CI (0.48–0.75), p < 0.001] using the random effects model for meta-analysis. Specifically, the Asp group demonstrated improved OS compared with that in the non-Asp group (**Figure 3A**).

To evaluate the survival benefit between the PEG-Asp and L-Asp groups, three studies were analyzed (7, 27, 28). Among the 174 patients included in the analysis, 97 and 56 were in the PEG-Asp and L-Asp groups, respectively. The random effects model revealed no statistical heterogeneity among studies (*p* = 0.39, I^2^ = 0%). The PEG-Asp group exhibited a lower HR than that of the L-Asp group [HR = 0.47, 95% CI (0.27–0.82), *p* = 0.007] (**Figure 3B**).

An analysis of three studies (19, 30, 31) revealed that among 147 patients newly diagnosed with ENKTCL, 84 and 63 belonged to the EBV DNA-positive and EBV DNA-negative groups, respectively. The EBV DNA-positive group before treatment exhibited poorer OS compared with that of the EBV DNA-negative group [HR = 2.57, 95% CI (1.10–6), *p* = 0.029] (**Figure 3C**).

Patients with R/R ENKTCL, who were possibly affected by EBV viremia, were included in the three studies (21, 31, 32). Among the 97 patients with R/R ENKTCL, 54 were EBV-positive at the end of the treatment, whereas 43 patients were EBV-negative. Significant differences were observed between the two groups [OR=9.71, 95% CI (2.36–39.88), *p*-value =0.002] (**Figure 3D**).

## 4 Discussion

### 4.1 Treatment of ENKTCL with L-Asp

ENKTCL exhibits pathologic characteristics such as blood vessel destruction, non-caseous necrosis, and atypical lymphocyte proliferation, leading to fever and elevated inflammatory cytokine levels (32). Anthracycline-based chemotherapies, such as CHOP (cyclophosphamide, hydroxydaunorubicin, oncovin, and prednisone), are ineffective owing to p-glycoprotein/MDR1 gene expression (33, 34). To overcome this, combining radiation therapy (>50 Gy) with non-anthracycline chemotherapy has shown superior OS in patients with limited-stage ENKTCL (35–37).

In advanced-stage ENKTCL, radiation field setting is challenging, making L-Asp-based chemotherapy crucial for improving survival outcomes. Cancer cells lack asparagine synthetase and require extracellular asparagine for survival. L-Asp depletes plasma asparagine, halting intracellular protein biosynthesis and killing lymphoma cells. Asp resistance in cancer cells is an adverse prognostic factor for patient outcomes (38). Survival outcomes of Asp-based chemotherapy surpass non-Asp-based chemotherapies, such as SMILE (dexamethasone, methotrexate, ifosfamide, L-Asp, and etoposide) (12, 39) and DDGP (dexamethasone, cisplatin, gemcitabine, and PEG-asparaginase) (7). Meta-analyses also support this trend. Additionally, PEG-Asp, synthesized to decrease the immunogenicity of the enzyme and prolong its half-life, demonstrated greater efficacy than L-Asp. Although limited studies have confirmed this, maintaining stable plasma asparagine depletion remains essential for inducing tumor-suppressive conditions. PEG-Asp showed reduced toxicity in grade 3–4 leukopenia and allergic reactions (7, 27, 28). A discordance was observed concerning the elevation of alanine aminotransferase and thrombocytopenia, possibly owing to the combination of different cytotoxic drugs administered. No studies have compared the prognostic index for natural killer cell lymphoma plus EBV (PINK-E) score between Asp and PEG-Asp. However, Wang et al. reported that PFS was generally superior in the PEG-Asp group among individuals with EBV viremia within normal levels than in those with elevated viremia levels. They observed better PFS in individuals under 60 years of age compared with those over 60 years. These variables were included in the PINK-E score (7).

In patients with R/R ENKTCL who did not receive L-Asp-based chemotherapy, L-Asp showed a similar PFS to that of advanced ENKTCL. However, the OS was shorter than that of patients with stages III–IV ENKTCL. These data recommend the use of an L-Asp-containing regimen in newly diagnosed patients and transitioning to newer agents when relapse occurs.

### 4.2 ENKTCL with EBV viremia

OS varied among patients with advanced-stage ENKTCL. For example, Li et al. reported a 3-year PFS rate of 32.42% (19), whereas Hu et al. observed a rate of 48.1% (10). This discrepancy may stem from different proportions of EBV DNA elevation in each cohort (**Table 1**). Our findings indicate that EBV DNA viremia predicts a poor prognosis. In newly diagnosed ENKTCL, pretreatment EBV viremia was a significant factor. These studies included patients with both limited and advanced-stage disease; therefore, this interpretation should be approached with caution. Yan et al. demonstrated that limited-stage disease with plasma EBV positivity had outcomes similar to those of stages III–IV (40). For R/R ENKTCL, patients with sustained EBV viremia post-treatment exhibited worse OS compared with that in those without sustained viremia. Thus, sustained viremia can serve as a surrogate marker for predicting relapse. In summary, the presence of EBV viremia requires careful consideration, and further treatment plans are necessary for high-risk ENKTCL.

Targeted therapies, including those using brentuximab, pembrolizumab and daratumumab, do not ensure complete treatment, and their sustained responses are limited (41–43). To address EBV viremia and achieve long-term survival, targeting the EBV antibody (LMP1/LMP2) with cytotoxic T lymphocyte therapy (CTL) has shown complete remission (44, 45). While autologous transplantation offers limited survival benefits, allogeneic hematopoietic stem cell transplantation has shown efficacy in advanced-stage III/IV and R/R ENKTCL. Combinations of CTL with either autologous or allogeneic HSCT may serve as a curative approach (46, 47).

Studies on advanced-stage or R/R ENKTCL are relatively scarce compared with those on limited-stage ENKTCL, and survival outcomes vary across studies. Therefore, we gathered and integrated data using graphs for this analysis. To the best of our knowledge, this is the first attempt to conduct meta-analyses on individual ENKTCL patient data. We compared survival outcomes and identified factors affecting them. However, our study has some limitations. First, we performed a meta-analysis on both prospective trials and retrospective studies. Nonetheless, our data provide valuable reference points for future prospective randomized controlled trials. Additionally, most of our data were estimated from graphs rather than documented data, posing a risk of human error during the extraction process. However, by imputing missing data using established methods, we increased the reproducibility of our findings.

In conclusion, our data indicate that L-Asp significantly improves outcomes in Ann Arbor stages III–IV and R/R ENKTCL. Furthermore, EBV viremia is a crucial target and tracking marker for predicting survival.

## Data availability statement

The data analyzed in this study is subject to the following licenses/restrictions: The data presented in this study are available on request from the corresponding author. Requests to access these datasets should be directed to Tong Yoon Kim, tyk@catholic.ac.kr.

## Author contributions

TYK: Data curation, Formal Analysis, Methodology, Visualization, Writing – original draft, Writing – review & editing. TJK: Resources, Writing – review & editing. EH: Data curation, Writing – review & editing. GM: Data curation, Writing – review & editing. YJ: Formal analysis, Writing – review & editing. S-GC: Conceptualization, Formal Analysis, Resources, Writing – original draft, Writing – review & editing.
