# Supplementary material for: Challenges in overcoming advanced-stage or relapsed refractory extranodal NK/T-cell lymphoma: meta-analysis of individual patient data
Source: Front Oncol. 2024 Jul 31;14:1362367. doi: 10.3389/fonc.2024.1362367 (PMC11322147; doi:10.3389/fonc.2024.1362367)
Supplement: Supplementary file 1 [file DataSheet_1.pdf]

Supplementary Material

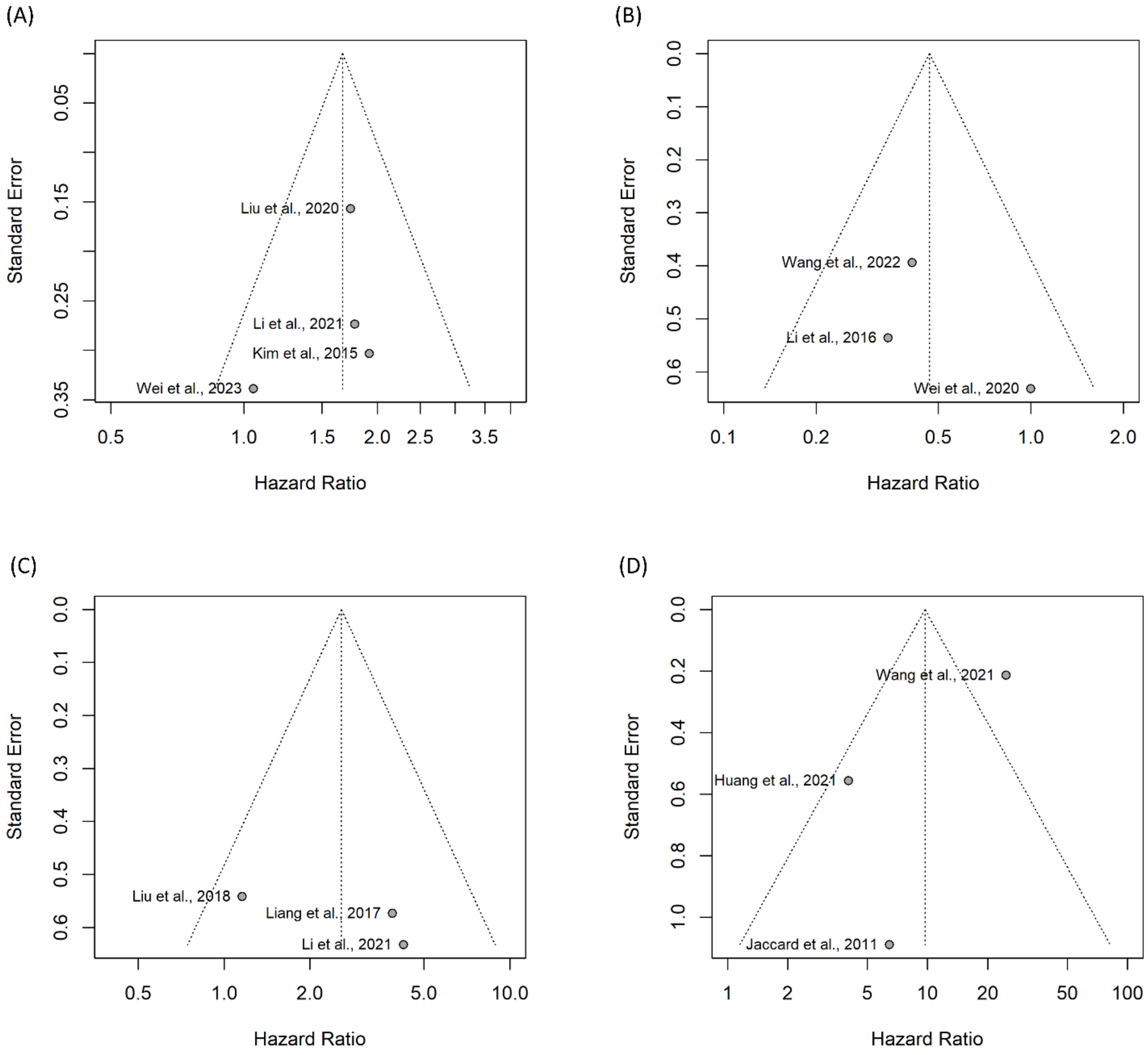

Supplementary Figure S1. Funnel plots of the included studies. (A) L-asparaginase (Asp)-based versus non-Asp-based chemotherapy. (B) Peg asparaginase compared with L-Asp-based chemotherapy in advanced-stage ENKTCL patients. (C) Elevated versus normal EBV DNA levels in pretreatment blood of stage I-IV ENKTCL patients. (D) Elevated versus normal EBV DNA levels in end-of-treatment blood of relapsed/refractory ENKTCL patients.
